# Study of the migration of *Fasciola hepatica* juveniles across the intestinal barrier of the host by quantitative proteomics in an *ex vivo* model

**DOI:** 10.1371/journal.pntd.0010766

**Published:** 2022-09-16

**Authors:** David Becerro-Recio, Judit Serrat, Marta López-García, Verónica Molina-Hernández, José Pérez-Arévalo, Álvaro Martínez-Moreno, Javier Sotillo, Fernando Simón, Javier González-Miguel, Mar Siles-Lucas

**Affiliations:** 1 Parasitology Unit, Institute of Natural Resources and Agrobiology of Salamanca (IRNASA-CSIC), Salamanca, Spain; 2 Departamento de Anatomía y Anatomía Patológica Comparadas y Toxicología, UIC Zoonosis y Enfermedades Emergentes ENZOEM, Facultad de Veterinaria, Universidad de Córdoba, Córdoba, Spain; 3 Departamento de Sanidad Animal (Parasitología), UIC Zoonosis y Enfermedades Emergentes ENZOEM, Facultad de Veterinaria, Universidad de Córdoba, Córdoba, Spain; 4 Centro Nacional de Microbiología, Instituto de Salud Carlos III, Majadahonda, Madrid, Spain; 5 Laboratory of Parasitology, Faculty of Pharmacy, University of Salamanca, Salamanca, Spain; 6 Molecular Parasitology Laboratory, Centre of One Health (COH), Ryan Institute, National University of Ireland, Galway, Ireland; University of Passo Fundo: Universidade de Passo Fundo, BRAZIL

## Abstract

*Fasciola hepatica* is a trematode parasite that infects animals and humans causing fasciolosis, a worldwide-distributed disease responsible for important economic losses and health problems. This disease is of growing public health concern since parasite isolates resistant to the current treatment (triclabendazole) have increasingly been described. *F*. *hepatica* infects its vertebrate host after ingestion of the encysted parasite (metacercariae), which are found in the water or attached to plants. Upon ingestion, newly excysted juveniles of *F*. *hepatica* (FhNEJ) emerge in the intestinal lumen and cross the intestinal barrier, reach the peritoneum and migrate to the biliary ducts, where adult worms fully develop. Despite the efforts made to develop new therapeutic and preventive tools, to date, protection against *F*. *hepatica* obtained in different animal models is far from optimal. Early events of host-FhNEJ interactions are of paramount importance for the infection progress in fasciolosis, especially those occurring at the host-parasite interface. Nevertheless, studies of FhNEJ responses to the changing host environment encountered during migration across host tissues are still scarce. Here, we set-up an *ex vivo* model coupled with quantitative SWATH-MS proteomics to study early host-parasite interaction events in fasciolosis. After comparing tegument and somatic fractions from control parasites and FhNEJ that managed to cross a mouse intestinal section *ex vivo*, a set of parasite proteins whose expression was statistically different were found. These included upregulation of cathepsins L3 and L4, proteolytic inhibitor Fh serpin 2, and a number of molecules linked with nutrient uptake and metabolism, including histone H4, H2A and H2B, low density lipoprotein receptor, tetraspanin, fatty acid binding protein a and glutathione-S-transferase. Downregulated proteins in FhNEJ after gut passage were more numerous than the upregulated ones, and included the heath shock proteins HSP90 and alpha crystallin, amongst others. This study brings new insights into early host-parasite interactions in fasciolosis and sheds light on the proteomic changes in FhNEJ triggered upon excystment and intestinal wall crossing, which could serve to define new targets for the prevention and treatment of this widespread parasitic disease.

## Author summary

Fasciolosis caused by the helminth parasite *Fasciola hepatica* is a serious health and economic problem worldwide. Treatment and prevention of this disease pose several drawbacks that have so far not been solved. The definition of suitable parasite molecular targets to overcome such drawbacks should be based on thoroughly deciphering host-parasite interactions, and in this regard most studies have focused on the adult stages of *F*. *hepatica*. Nevertheless, in this context, the study of the transient juvenile stages of this parasite could be of higher utility due to the importance of early interactions with the host for parasite migration and the successful establishment of infection. In this work, we set-up an *ex vivo* model and performed a quantitative proteomics approach to study the changes in *F*. *hepatica* juveniles upon gut passage. We found that the parasite tegument and somatic compartments experienced deep changes in their composition and showed that the host triggers the expression of specific molecules that are important for parasite migration and survival at this stage. The molecules described here could serve to better understand host-parasite interactions and to define new targets against fasciolosis.

## Introduction

*Fasciola hepatica* is responsible for most cases of fasciolosis, which is considered as the foodborne parasitic disease with the widest geographical distribution [1] as a result of the ability of *F*. *hepatica* to persist in different ecosystems and to develop in a wide variety of intermediate and definitive hosts [2]. Economic losses derived from this disease are estimated to exceed $3 billion per year [3] and mainly affect ruminant livestock, arising from reduced production of animal by-products as well as an increased susceptibility to other diseases. Fasciolosis is also considered a major food-borne zoonotic disease as human cases have been reported in up to 51 countries [4]. Current estimations suggest that around 2.6 million people could be affected by this disease, and up to 90 million may be at risk of infection [5].

The life cycle of *F*. *hepatica* in the vertebrate host starts upon ingestion of raw aquatic plants or water contaminated with metacercariae, which rapidly excyst in the host duodenum and release the newly excysted juvenile flukes (FhNEJ). The first contact between the parasite and host tissues occurs at the intestinal level, from where FhNEJ start migrating by crossing the host’s intestinal wall in around 2–3 hours after excystment [6]. This mechanism could be considered as the “point of no return” in fasciolosis in terms of disease progression, since it represents the first step of an intricate migratory route followed by FhNEJ that eventually drives them towards the major intra-hepatic biliary ducts, a location that is poorly accessible to effectors of the host immune response [7].

Despite the efforts to obtain a better understanding of host-parasite relationships in fasciolosis carried out over the past decades, relatively little is known about the molecular cross-talk between *F*. *hepatica* and its hosts during the early stages of infection. In order to fill this knowledge gap, different models have been developed to study the molecular milieu at the FhNEJ-host interface by using *ex vivo* [8] or *in vitro* [9] experimental rat models that mimic the passage through the intestine by FhNEJ. In this line, our group recently developed an *in vitro* model aimed at replicating the early contact between FhNEJ and the intestinal epithelium of the host [10]. In this model, *F*. *hepatica* metacercariae were excysted *in vitro* and placed over a primary cell culture representing the mouse intestinal epithelium. After 24 hours of co-incubation, both FhNEJ and cells were subjected to proteomic analysis using Isobaric Tag for Relative and Absolute Quantitation (iTRAQ) to determine which proteins were differentially expressed in both species during their interaction. This strategy resulted in the identification of 191 and 62 up-regulated, and 112 and 57 down-regulated proteins in the FhNEJ tegument and somatic extracts, respectively. Similarly, 87 up-regulated and 73 down-regulated proteins in the extract of host cells were identified. Regulated proteins were related to parasite development, invasion and evasion, as well as manipulation of the host intestinal epithelial cell adhesion, immunity and apoptosis pathways, amongst others. -Omics approaches can be useful not only to investigate the changes arisen in the host-parasite interface but also to find new and effective therapeutic targets against fasciolosis [11]. In this context, the development of innovative and more accurate proteomic approaches such as Sequential Window Acquisition of All Theoretical Mass Spectra (SWATH-MS) could provide an improvement in terms of reproducibility and sensitivity [12–15].

In this work, we set-up an *ex vivo* model to replicate the passage of FhNEJ through the intestinal wall of the host using a murine model combined with SWATH-MS analysis to understand how the expression profile of FhNEJ is modified during this process, which reveals a list of candidate proteins that might be relevant during the intestinal stage of *F*. *hepatica* infection.

## Materials and methods

### Ethics statement

All animals received humane care in conformity with the Directive for the protection of animals utilized for scientific purposes (Directive 2010/63/UE, Decision 2020/569/UE and RD 1386/2018). All methods have been authorized by the Ethical Animal Experimentation Committee of the University of Córdoba and by the Junta de Andalucía (project no. 2021PI/22).

### *F*. *hepatica in vitro* excystment

A total of 10,000 metacercariae from the *F*. *hepatica* Italian strain were purchased from Ridgeway Research LTD (UK), and *in vitro* excystment was performed according to previous reports [16]. Briefly, CO_2_ was bubbled into a sterile tube containing 10 ml of distilled (d)H_2_O for 30 s, and sodium dithionite was added to a final concentration of 0.02 M. The tube was incubated at 37°C for 5 min, the mix was added to metacercariae and incubated at 37°C for 1 hour. After incubation, metacercariae were washed twice with warm dH_2_O and resuspended in 10 ml of excystment medium consisting of 0.03 M HEPES, 10% rabbit bile (from a local abattoir) and Hank’s balanced salt solution. Metacercariae were distributed in a 6-well plate, incubated at 37°C for 4 h and the excystment process was monitored every hour under a microscope. While FhNEJ emerged, empty metacercariae cysts and unexcysted metacercariae were removed using a micropipette. Around 1,500 FhNEJ were collected to be used as negative controls (see *Ex vivo* model section), while the rest were centrifuged (5 min, 300 x *g*) and left in 200 μl of supernatant (containing the FhNEJ), which was reserved for further use.

### *Ex vivo* model

**Fig 1:**
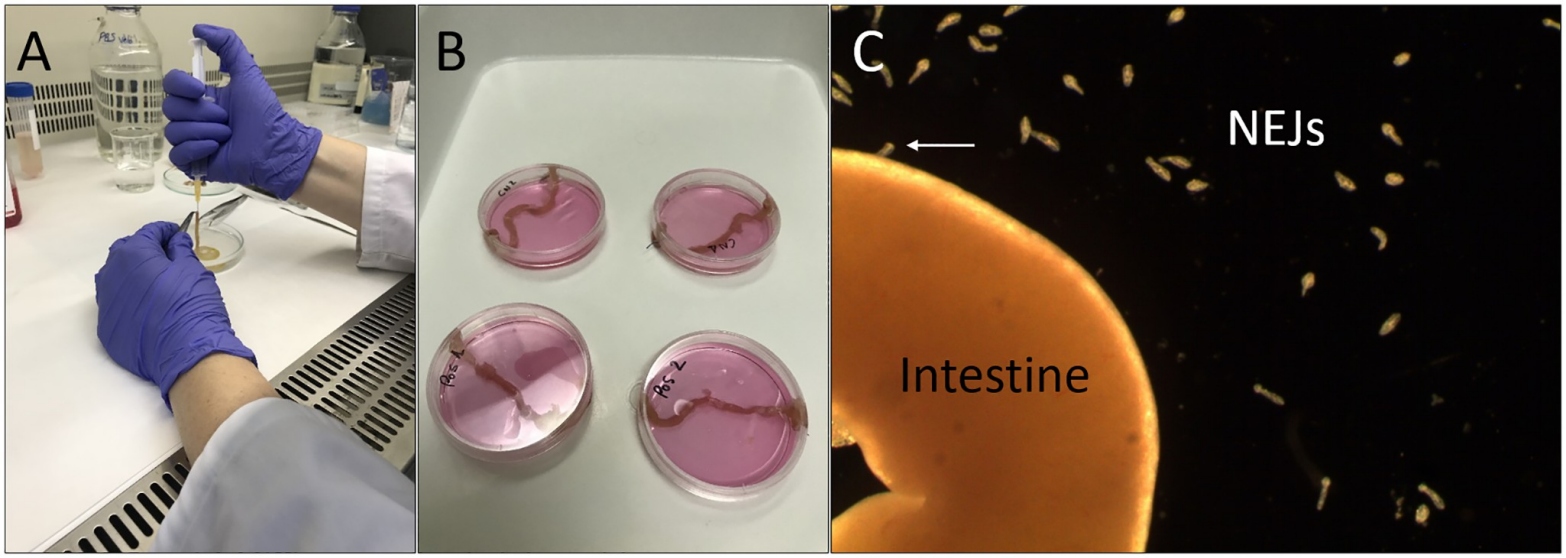
*Ex vivo* model for the migration of FhNEJ through mouse intestine. A) Preparation of the intestines before FhNEJ infection; B) Incubation of ligated intestines in RPMI medium at 39°C 5%CO_2_ for 2.5 h; C) Image obtained in a stereomicroscope showing the FhNEJ after intestinal passage (white arrow).

A total of nine 12 week old male C57Bl/6 mice were housed by the Central Service of Experimental Animals at the University of Córdoba and divided in 3 replicates. The experimental procedure of the *ex vivo* model began with a 24 h period of fasting prior to necropsy. The day of the experiment, mice were euthanized by CO_2_ overdose and cervical dislocation, and the stomach and the subsequent 15 cm of the small intestine were collected to perform the *ex vivo* assay. Intestines were cut close to the pylorus to remove the stomach and only the duodenum and jejunum portions were kept. Intestinal contents were flushing out under a laminar flow hood by two gentle washes with a 5 mL syringe coupled to 21G needles containing sterile phosphate-buffered saline (PBS) (Fig 1A). One end of the intestines was firmly ligated and a solution of 200 μl of excystment medium, containing an average of 2,500 FhNEJ per intestine, was pipetted inside the intestinal lumen through the caudal section of the intestine, which was immediately ligated after FhNEJ addition. Intestines injected with excystment medium devoid of FhNEJ were used as negative controls. Both experiment (injected with FhNEJ) and control intestines were placed in 60 mm petri dishes containing RPMI medium (leaving the ligated ends outside the plate), which were incubated for 2.5 hours at 39°C and 5% CO_2_ (Fig 1B). Additionally, untreated intestines were placed in fixative immediately after collection and washed as described in the section “Histopathological and immunohistochemical study”. The plates were examined under a stereomicroscope before and after incubation to visualize the appearance of FhNEJ outside the intestines. The *ex vivo* assay was performed in triplicate. FhNEJ used as negative controls were incubated alone after excystment in petri dishes (each one with 500 FhNEJ) containing RPMI medium for 2.5 hours at 39°C. After incubation, FhNEJ that crossed the intestinal walls, and those used as negative controls, were manually collected with a micropipette and washed twice with sterile PBS, centrifuged at 300 x *g* for 5 min and immediately subjected to protein extraction.

### Protein extraction

In order to study *F*. *hepatica* tegument and somatic proteins separately, tegument extraction of FhNEJ samples was performed as previously described [9]. Briefly, FhNEJ were resuspended in 500 μl sterile PBS containing 1% Nonidet P-40 and incubated at room temperature with soft stirring (60 rpm using a rotatory mixer) for 30 min. FhNEJ were then centrifuged (300 x *g* for 5 min) and the supernatant containing the tegument protein fraction was stored at -80°C until use. The resulting pellet was resuspended in 500 μl RIPA lysis buffer and disrupted by ultrasonic pulsing (5 cycles of 30 s, leaving the samples on ice between cycles to avoid overheating) to release the somatic fraction. Finally, protein samples were centrifuged (1,000 x *g* for 5 min) and the supernatant containing the somatic proteins was stored at -80°C. All samples were treated with the Protease Inhibitor Cocktail (Sigma) at 1x to avoid protein degradation.

### Mass spectrometry analysis

Protein samples were quantified using a detergent compatible kit (Protein Quantification Assay; Machery-Nagel) following the manufacturer’s instructions. Protein samples were in-gel digested as previously described [17] with slight modifications: 20 μg of each sample was resuspended in 20 μl of Laemmli Sample Buffer (Bio-Rad) and denatured at 95°C for 5 min, after which they were loaded onto an Any kD precast 1D PAGE gel (Bio-Rad) and run at 200 V for 5 min. After separation, proteins were fixed with 40% ethanol/10% acetic acid. Each lane of the gel was cut into pieces and treated with reducing and alkylating agents (dithiothreitol and iodoacetamide, respectively), after which the proteins were overnight digested with sequencing grade trypsin (Promega). The digestion was stopped with 1% trifluoroacetic acid (TFA) and the resulting peptides were extracted with acetonitrile (ACN). Finally, each sample was dried on a rotary evaporator and resuspended in 20 μl of 2% ACN; 0.1% TFA.

For library construction, all samples were pooled and loaded onto an analytical column (LC Column, 3 μ C18-CL, Nikkyo) equilibrated in 5% ACN 0.1% formic acid. Peptides were eluted in a linear gradient of 5–35% solvent B (A: 0.1% FA; B: ACN, 0.1% FA) over 120 min at 300 nl/min flow rate and analyzed in a mass spectrometer nanoESI qQTOF (5600 TripleTOF, ABSCIEX). Analysis was carried out in a data-dependent mode (DDA). Survey MS1 scans were acquired from 350–1250 m/z for 250 ms, whereas the quadrupole resolution was set to “UNIT” for MS2 experiments, which were acquired 100–1500 m/z for 150 ms in high sensitivity mode.

For individual sample acquisition, the tripleTOF was operated in SWATH mode (DIA), in which a 0.050-s TOF MS scan from 350–1250 m/z was performed, followed by 0.080-s product ion scans from 350–1250 m/z on the 32 defined windows (3.05 sec/cycle).

### Database search and bioinformatics analysis

Protein Pilot v5.0 (SCIEX) was used to generate a peak list directly from 5600 TripleTof.*wiff* files corresponding to the peptide library. The database used contained the predicted proteome of *F*. *hepatica* (PRJEB25283, https://parasite.wormbase.org/Fasciola_hepatica_prjeb25283/ Info/Index), appended to the cRAP contaminant database (https://www.thegpm.org/crap/). The Paragon algorithm [18] was applied to the database with the following parameters: trypsin specificity, IAM cys-alkylation, taxonomy no restricted. Only proteins with at least 2 identified peptides and < 1% FDR were considered for subsequent analysis.

The.*wiff* files obtained from the SWATH experiment were analyzed using PeakView 2.1 (SCIEX) and MarkerView 3.0 (SCIEX). Protein areas were normalized by the total sum of the areas of all the quantified proteins, and proteins matching the contaminant database were removed from the dataset prior to differential expression analysis.

Principal Component Analysis (PCA) of all samples was conducted with the online tool ClustVis [19] and statistical evaluation was performed using GraphPad Prism 9.0. Data was Log2 transformed prior to differential expression analysis, and differences between control and incubated samples were determined by Student’s *t*-Test. *P* values were adjusted using the Benjamini, Krieger and Yekutieli post-hoc corrections. Differentially expressed proteins were identified by q value < 0.05.

Changes in the expression profile within the two analyzed compartments (tegument and soma) were represented in Volcano plots using the ggplot2 package of R software. Differentially expressed proteins were analyzed using Blast2GO 5.2 in order to obtain the associated Gene Ontology (GO) terms in the Biological Process, Molecular Function and Cellular Component categories, and the most enriched GO terms were visualized using the WeGO 2.0 tool (http://wego.genomics.cn/) [20] and the REVIGO tool (http://revigo.irb.hr/) [21]. Unidentified proteins were manually identified using the NCBI blastp algorithm.

### Immunoblotting

Protein samples used in mass spectrometry analysis were first concentrated using centrifugal filters with a nominal molecular weight limit of 3 kDa (Merck Millipore) by centrifuging 30 min at 5,000 x *g* and 4°C and protein concentrations were measured using the Pierce BCA Protein Assay (Thermo Scientific). Equal amounts of protein were separated by SDS-PAGE in 12% gels and blotted onto nitrocellulose membranes. After transfer, total protein staining was performed with SYPRO Ruby (Thermo Fisher) according to manufacturer’s instructions and membranes were blocked in PBS-0.05% Tween containing 2% BSA. The primary antibody anti-*Fasciola hepatica* serpin 2 (FhSrp2) produced in rabbit against the recombinant protein rFhSrp2 of *F*. *hepatica* [22] was diluted 1:500 in blocking buffer and added to the blot overnight at 4°C. After incubation with anti-rabbit horseradish peroxidase (HRP)-conjugated secondary antibody (diluted 1:2,000 in blocking buffer; Sigma), the bands were detected using enhanced chemiluminiscence (Clarity Western ECL Substrate, Bio-Rad) on a ChemiDoc MP Imaging System (Bio-Rad).

### Histopathological and immunohistochemical study

For the histopathological study, a solution of 10% buffered formaldehyde was injected into the intestinal lumen and the intestines were immersed in the same fixative solution for 24 h. Next, intestines were sequentially trimmed in sections of 0.3 cm and embedded in paraffin wax, sectioned (4 μm thick) and stained with haematoxylin/eosin. For immunohistochemistry, sections (3 μm thick) were dewaxed, rehydrated and endogenous peroxidase activity was exhausted by incubation with 0.3% hydrogen peroxide in methanol for 30 min at room temperature. Antigen retrieval was performed by heating samples for 10 min in 0.01 M sodium citrate buffer (pH 6). Sections were washed in PBS (pH 7.2) and incubated with 20% normal goat serum (Vector Laboratories, Burlingame, California, USA) for 30 min at room temperature. Rabbit anti-mouse Caspase-3 (Biorbyt) primary antibody was diluted 1:400 in PBS containing 10% normal goat serum and incubated overnight at 4°C. Following washing in PBS, sections were incubated with goat anti-rabbit biotinylated secondary antibody (Dako) diluted 1:200 in PBS containing 10% normal goat serum for 30 min at room temperature. After washing in PBS, all sections were incubated with the ABC complex (Vectastain ABC Elite Kit) for 1 h at room temperature in darkness, washed in 0.05 M Tris buffered saline (pH 7.6) and then incubated with the chromogen solution (Vector NovaRED Peroxidase Substrate Kit). Finally, all sections were counterstained with Harris’ hematoxylin and mounted with Eukitt (Freiburg, Germany). As a negative control, the specific primary antibody was substituted with non-immune isotype-matched sera.

## Results

### *Ex vivo* model

The *ex vivo* experimental system herein described allowed us to successfully replicate the passage of FhNEJ through the intestinal wall of the vertebrate host using a murine model. First, FhNEJ were excysted *in vitro* and collected as they emerged from the metacercariae, resulting in an excystment rate of 80% after 3 hours of *in vitro* incubation. Viability of excysted parasites was checked under a microscope, and only actively moving juveniles were used for the *ex vivo* experiment. By periodically monitoring the passage of parasites under the microscope, we observed that most of FhNEJ crossed the intestinal wall within the first 30 minutes after injection into the intestinal lumen. By the end of incubation (2.5 hours later), approximately 22% of the total FhNEJ injected had managed to break through the intestine (Fig 1C and S1 Video).

### Protein identification and quantification

Proteins extracted from the tegument and soma of control parasites and FhNEJ that crossed the intestinal wall were subjected to SWATH-MS. A data-dependent acquisition approach was performed to identify the proteins contained in the library constructed from the pool of all samples. A total of 6,169 mass spectra were obtained and data deposited to the proteomeXchange Consortium via the PRIDE [23] partner repository with the dataset identifier PXD033945. The mass spectra corresponded to 3,384 unique peptides using a local FDR < 1%. These peptides represented 541 different proteins containing two or more unique peptides. Of these, 53 were found in the contaminant database and were not considered for further analyses.

**Fig 2:**
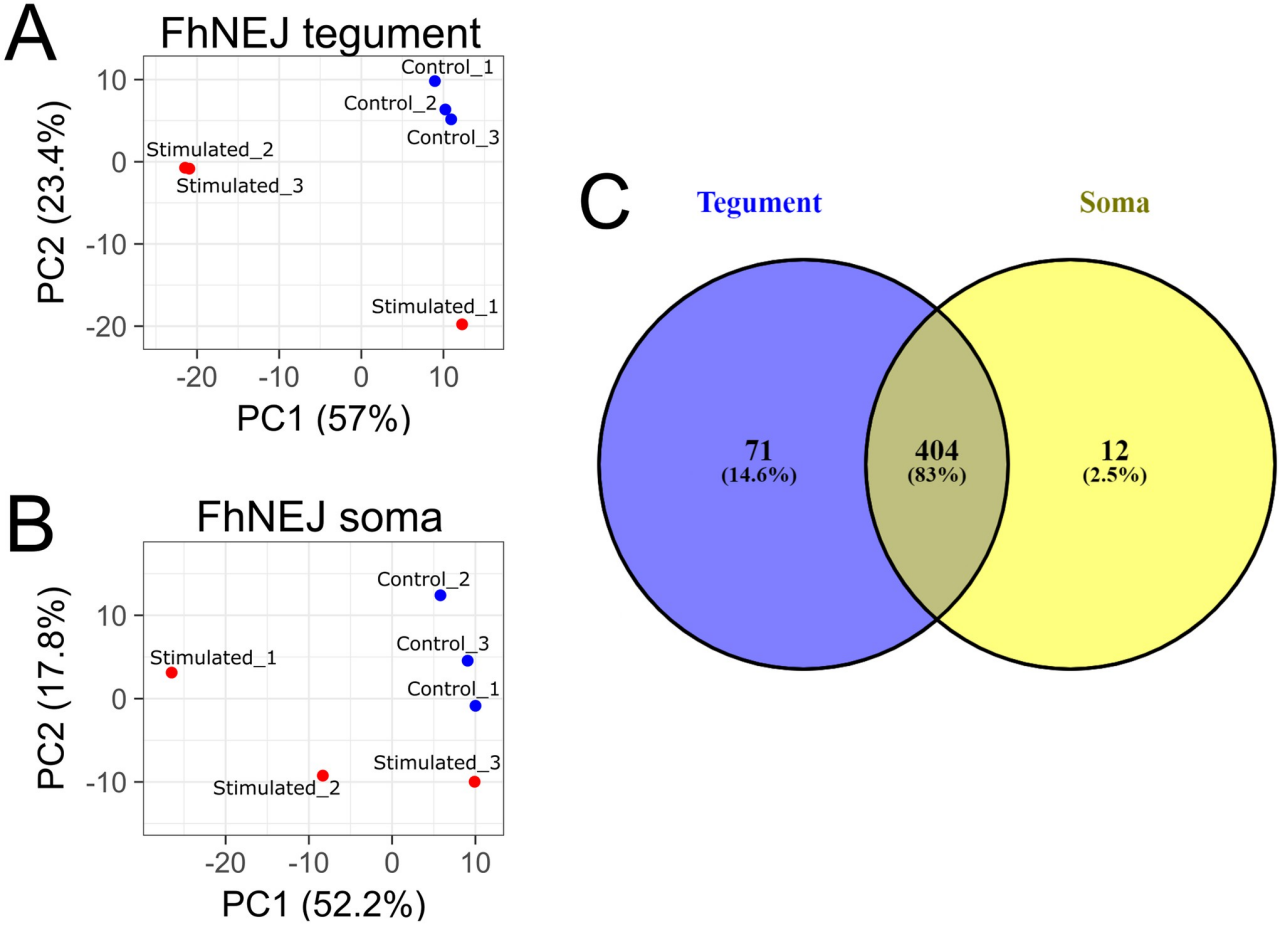
Principal Component Analysis (PCA) of FhNEJ (A) tegument and (B) somatic extracts. Blue dots represent the control replicates (Control_1 to _3) whereas red dots represent the replicates of FhNEJ after migration across the gut wall (Stimulated_1 to _3). The percentage of variance showed by each principal component (PC1 and 2) is indicated in its corresponding axis. (C) Venn’s diagram showing the distribution of quantified proteins.

Principal Component Analysis (PCA) of the FhNEJ tegument and somatic extracts was performed for the three replicates of each experimental condition. PC1 was the highest contributor to the variance of both tegument and somatic extracts (Fig 2A and 2B). For tegument extracts, control samples showed a close clustering, and samples from FhNEJ after gut passage showed some variation in the PC1 component, although they were clearly separated from control samples by either PC1 or PC2 variation (Fig 2A). Similar results were obtained for somatic extracts, although samples for each condition showed less optimal clustering as compared to their tegument counterparts (Fig 2B).

**Fig 3:**
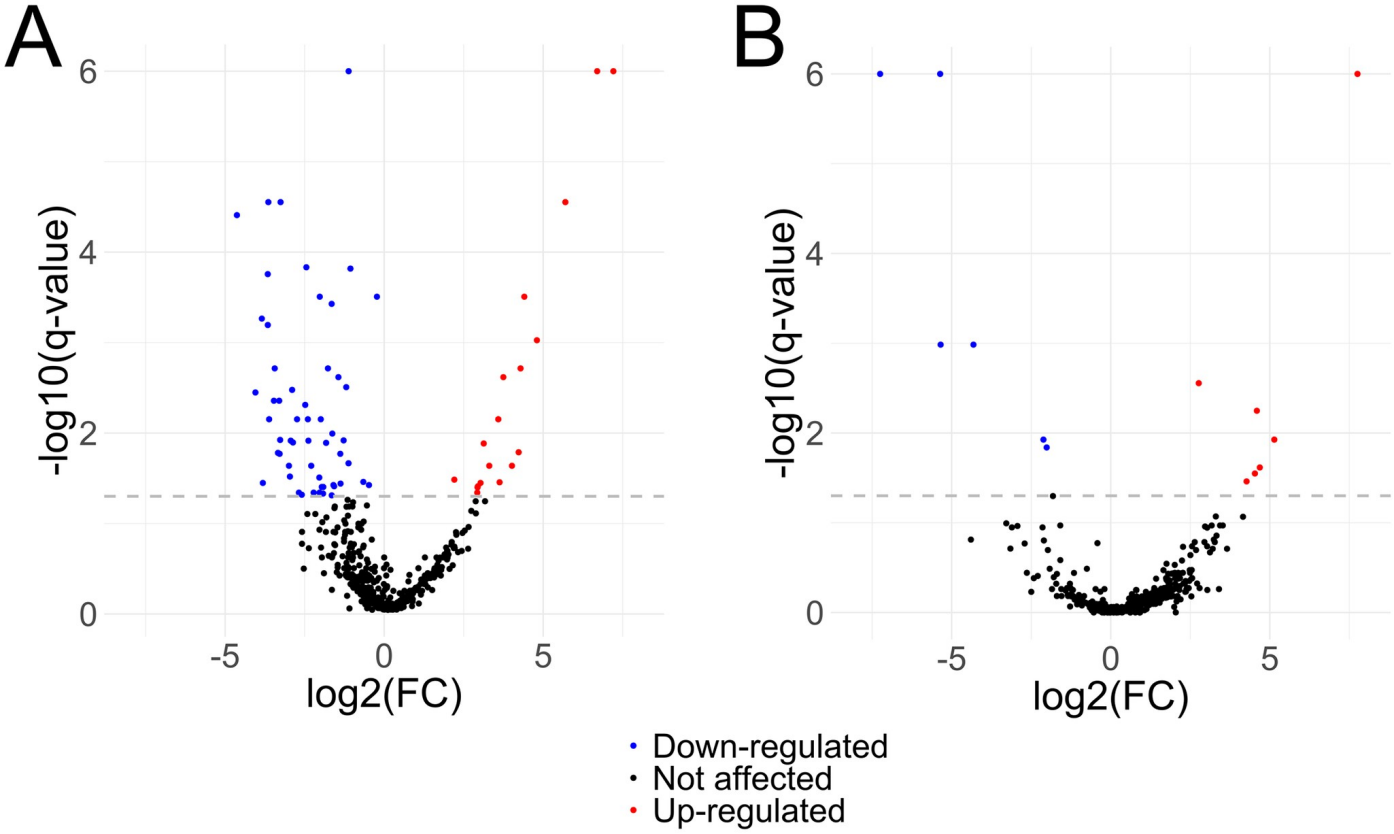
Volcano plots of the significantly differentially expressed proteins in tegument (A) and somatic extract (B) of *F*. *hepatica* juveniles after passing through mouse intestinal wall. The grey dashed line represents the threshold delimiting the differentially expressed proteins. Red dots represent upregulated proteins, while blue dots represent downregulated proteins.

After SWATH-MS analysis, a total of 475 proteins were detected in all tegument samples and could therefore be quantified, while 416 proteins were identified in all somatic samples. 404 of those proteins (83% of total quantified proteins) were common to both compartments, while 71 and 12 proteins, representing 14.6% and 2.5% of their total quantified proteins, were uniquely detected in tegument and soma, respectively (Fig 2C). Statistical analysis of the SWATH-MS data from FhNEJ recovered after crossing the mouse intestine revealed 18 upregulated and 54 downregulated proteins in the tegument fraction, whereas 7 upregulated and 6 downregulated proteins were found in the soma, in comparison with control FhNEJ (Fig 3A and 3B, respectively).

### Functional annotation

**Fig 4:**
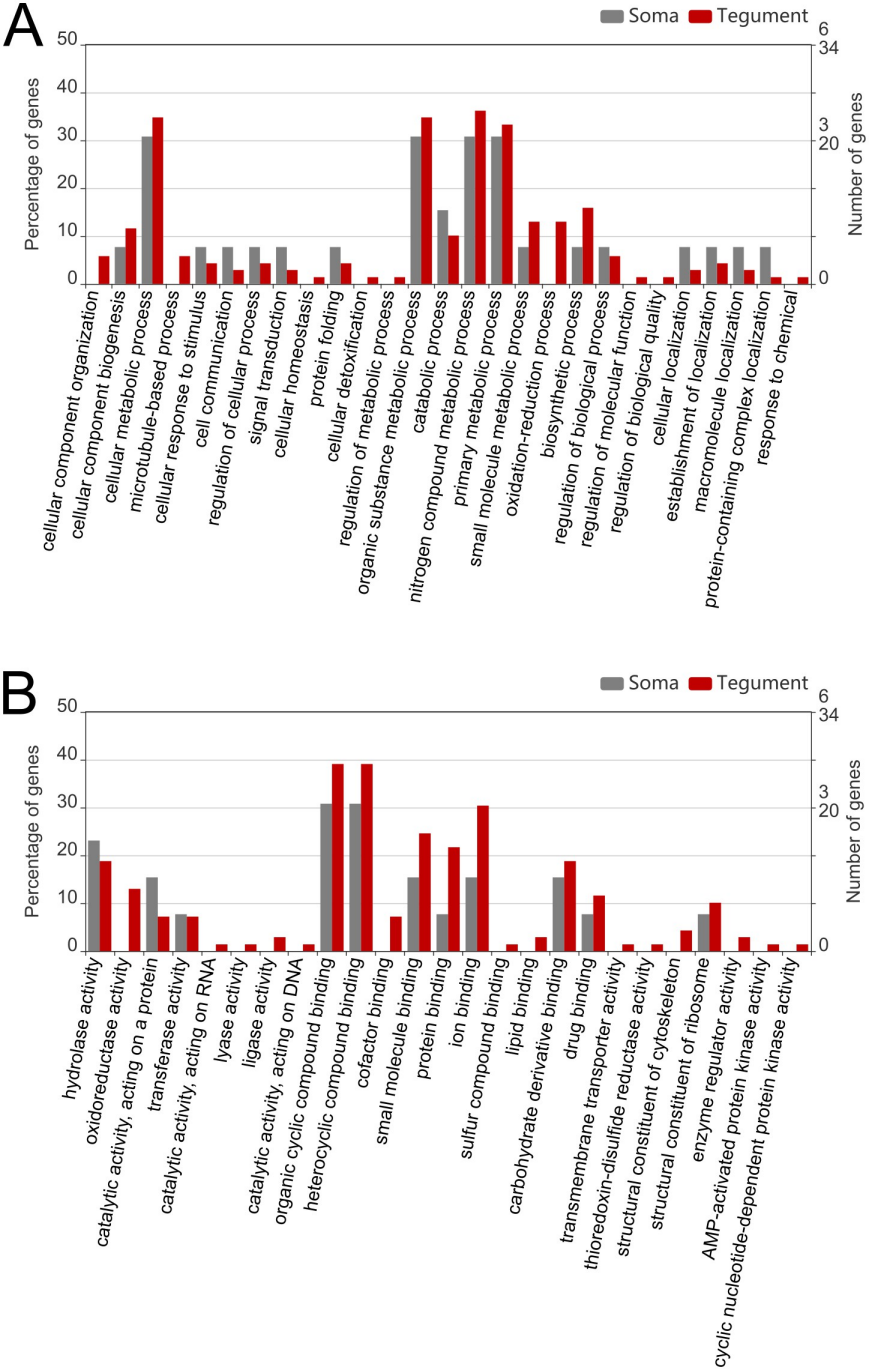
Bar graphs representing the results of the Gene Ontology analysis performed on the differentially expressed proteins, referred to Biological Process (A) and Molecular Function (B) categories. Red bars represent the proportion of proteins found in the tegument samples, and grey bars represent the proportion of proteins found in the somatic samples.

Gene Ontology annotation analysis (in the categories of Biological Process–BP–and Molecular Function–MF–) was performed on the differentially expressed proteins, and the results were plotted using the WEGO 2.0 tool (Fig 4A and 4B, respectively). In the tegument of FhNEJ, the main GO terms identified included terms mainly related to metabolic and biosynthetic processes in the BP category, and mainly related to binding functions in the MF category (Fig 4). REVIGO analysis of these extracts showed that some of the most representative terms in the BP category were linked with proteolysis, transcription and glutathione metabolic process within upregulated proteins, and within the tricarboxylic acid cycle and mechanisms of cellular oxidant detoxification within down-regulated proteins (S1 Fig).

**Fig 5:**
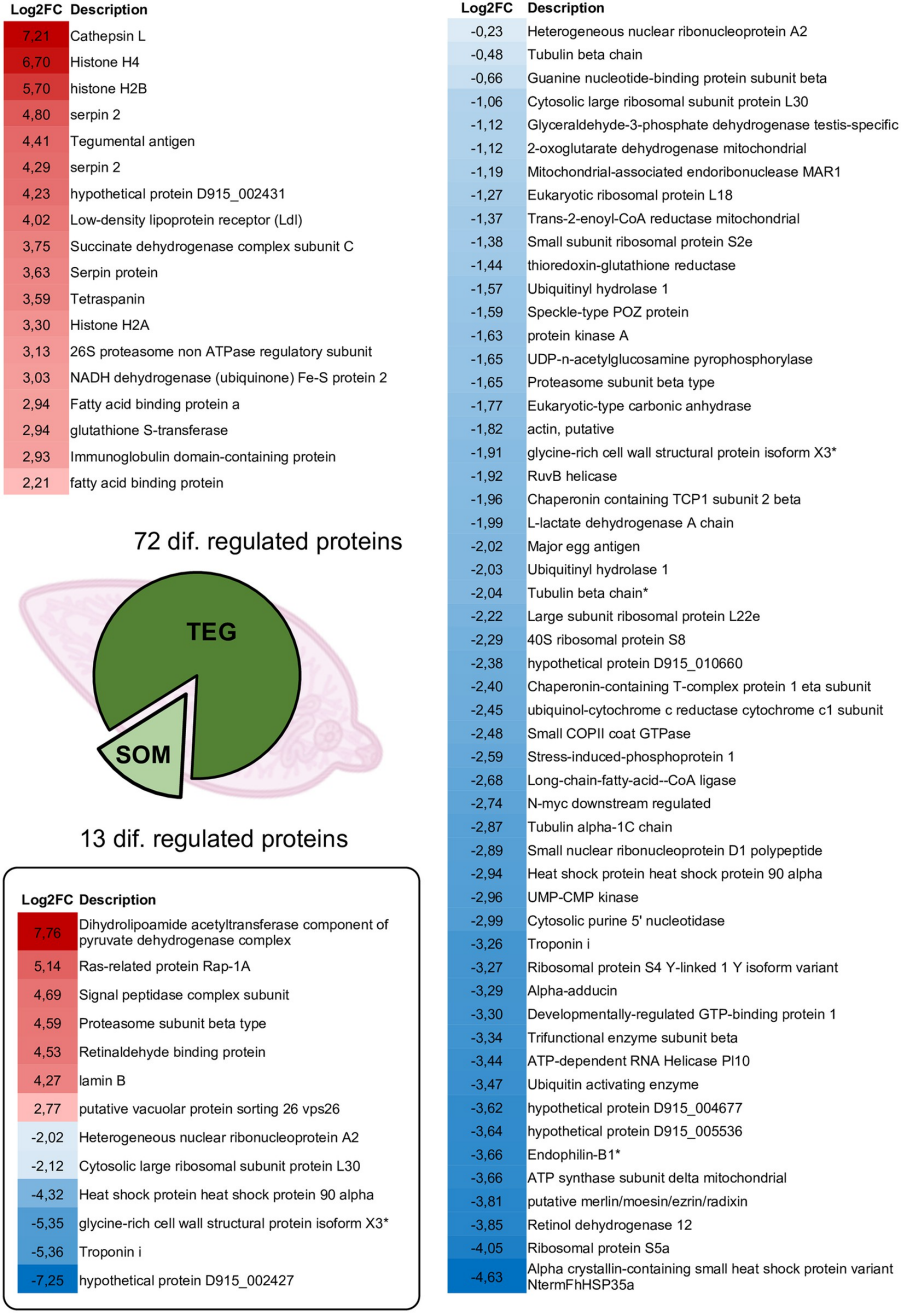
Upregulated (in red) and downregulated (in blue) proteins in tegument (upper panels) and somatic (box lower panel) extracts of *F*. *hepatica* juveniles after intestinal passage, compared with control FhNEJ. Both protein description and the respective Log2FC values are shown. Colour scales represent the relative Log2FC value in each panel.

Details on annotation and analysis of upregulated and downregulated proteins in both tegument and somatic extracts are shown in Fig 5 and S1 Table. The topmost upregulated protein in the tegument was the protease cathepsin L (Log2FC 7.21), followed by histones H4 and H2B and the protease inhibitor Fh serpin 2. For the histones identified in *F*. *hepatica* extracts, the mass spectra were compared with the corresponding proteins from both *F*. *hepatica* and *Mus musculus*, and the identifiers assigned as preferred were those of *F*. *hepatica*, so despite being very conserved molecules, we could unequivocally assign those identifications to the parasite. Serpin protease inhibitors were overexpressed in the tegument extract with three different UniProt identifiers (see S1 Table) corresponding to three different proteins, and the sum of their respective Log2FC values place serpins in the first position of overexpressed proteins in the tegument of FhNEJ after gut passage. Overexpressed proteins in the tegument of FhNEJ upon gut passage also included a tegument antigen, the hypothetical protein D915_002431 containing a sperm-protein, enterokinase and agrin domain [24], and a low-density lipoprotein (LDL) receptor, followed by two components of the electron transport chain, tetraspanin, two fatty acid binding proteins and the glutathione S-transferase Mu-class, among others (Fig 5).

Among downregulated tegument proteins, we identified numerous proteins related to peptide synthesis and degradation together with proteins with antioxidant activity and metabolic enzymes (Fig 5). The top ten downregulated proteins in terms of Log2FC included alpha crystallin, ribosomal protein 55a, retinol dehydrogenase 12 and endophilin B1, among others (S1 Table).

In the somatic fraction of FhNEJ that crossed the intestinal wall, the most represented GO terms were also related to metabolic processes in the BP category, and with binding and catalytic activity in the MF category (Fig 4). REVIGO analysis showed that BP terms related to glycolysis and gluconeogenesis were enriched in upregulated proteins, while downregulated proteins were mostly related to ribosome biogenesis, mRNA processing and protein folding (S1 Fig).

Accordingly, upregulated somatic proteins (Fig 5 and S1 Table) included enzymes involved in proteasomal degradation and protein secretion, together with vesicle trafficking related proteins, while downregulated proteins were mainly involved in protein synthesis and folding, as well as in mRNA processing. Noteworthy, five out of six proteins found downregulated in the somatic extract behaved likewise in the tegument extract.

### Immunoblot

**Fig 6:**
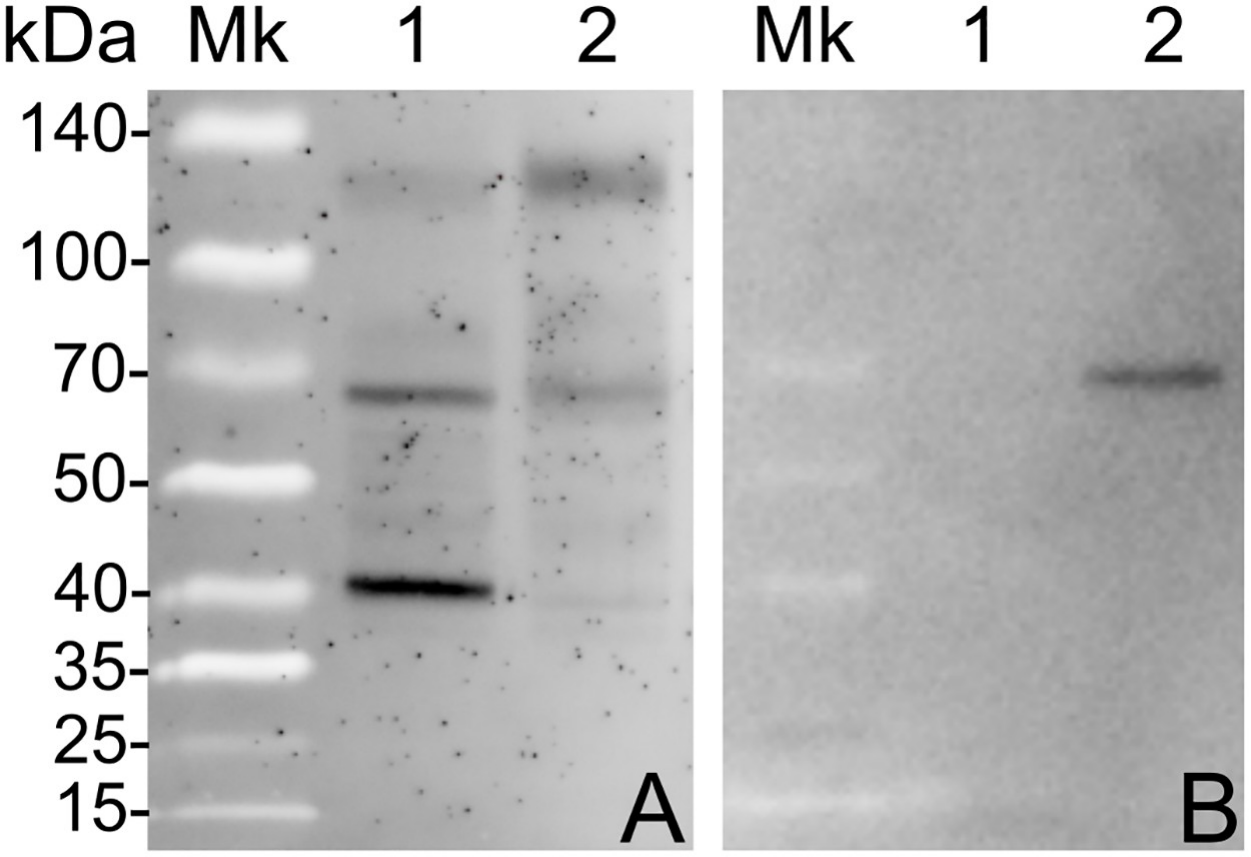
SDS-PAGE (A) and immunoblot (B) of tegument extracts from control FhNEJ (1) and FhNEJ after intestinal passage (2). (A) Sypro staining of total proteins; (B) anti-rFhSrp2 immunoblot. Molecular weights are shown in kDa.

In order to validate our model, tegument extracts from control FhNEJ and FhNEJ that crossed the intestinal wall were subjected to SDS-PAGE and immunoblot using antibodies against recombinant *F*. *hepatica* serpin 2, rFhSrp2 [22]. Reactivity was detected at ~60 kDa in the tegument of FhNEJ after intestinal passage, while no reactivity was detected in tegument extracts of control FhNEJ (Fig 6).

### Immunohistological analysis of the intestine

**Fig 7:**
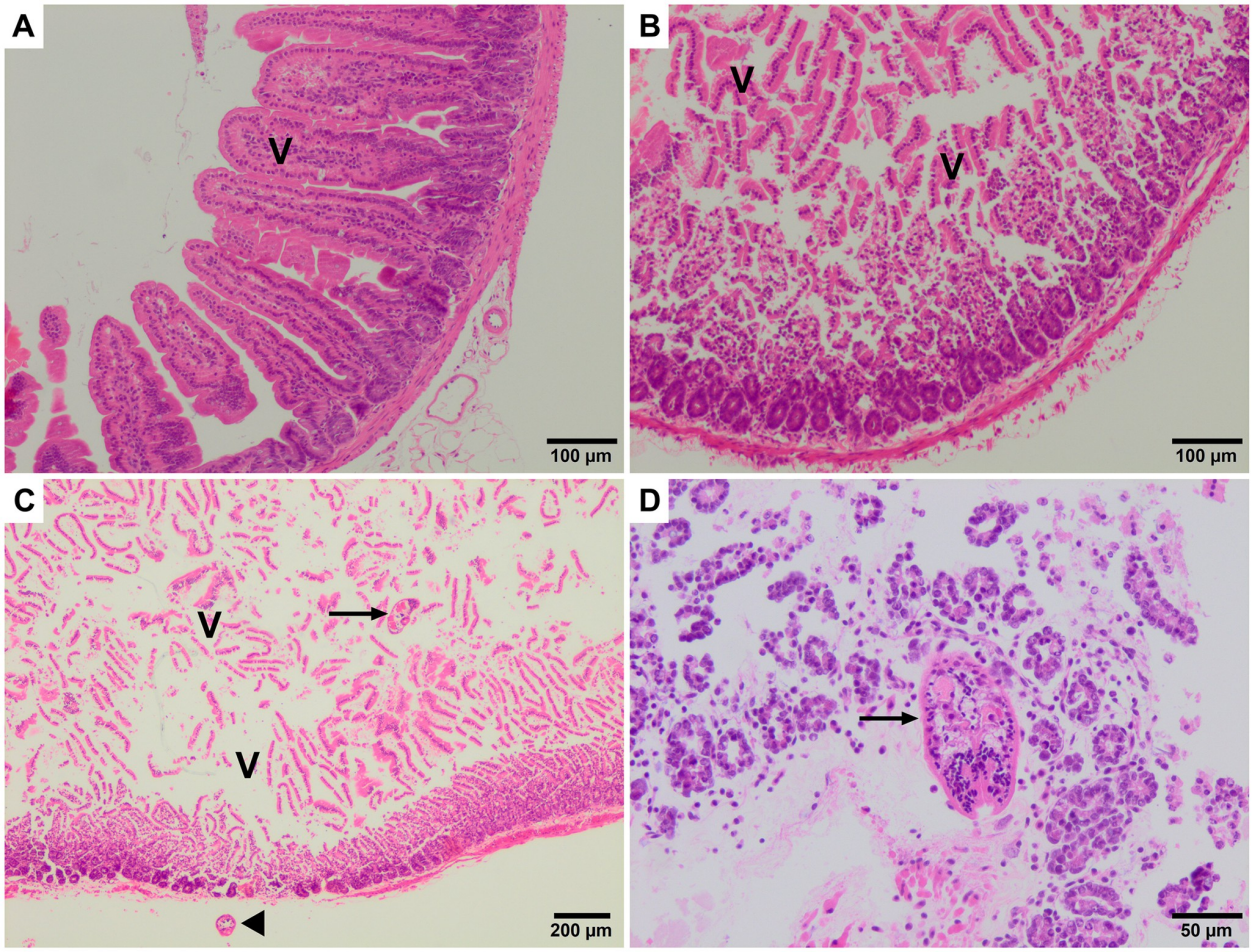
Histological features of the intestine of mice after hematoxylin-eosin staining. (A) Fresh intestine from a non-infected mouse showing normal villi (V). (B) Non-infected intestine after incubation for 2.5 hours at 39°C and 5% of CO_2_ showing detachment of villi (V). (C) Infected intestine after incubation and FhNEJ passage showing detached villi (V) and FhNEJ in the intestinal lumen (arrow) and in the intestinal serosa (arrowhead), both without inflammatory infiltrate. D) Detail of a FhNEJ (arrow) crossing the intestinal crypts. Note that there is no inflammatory infiltrate in the periphery of the FhNEJ.

**Fig 8:**
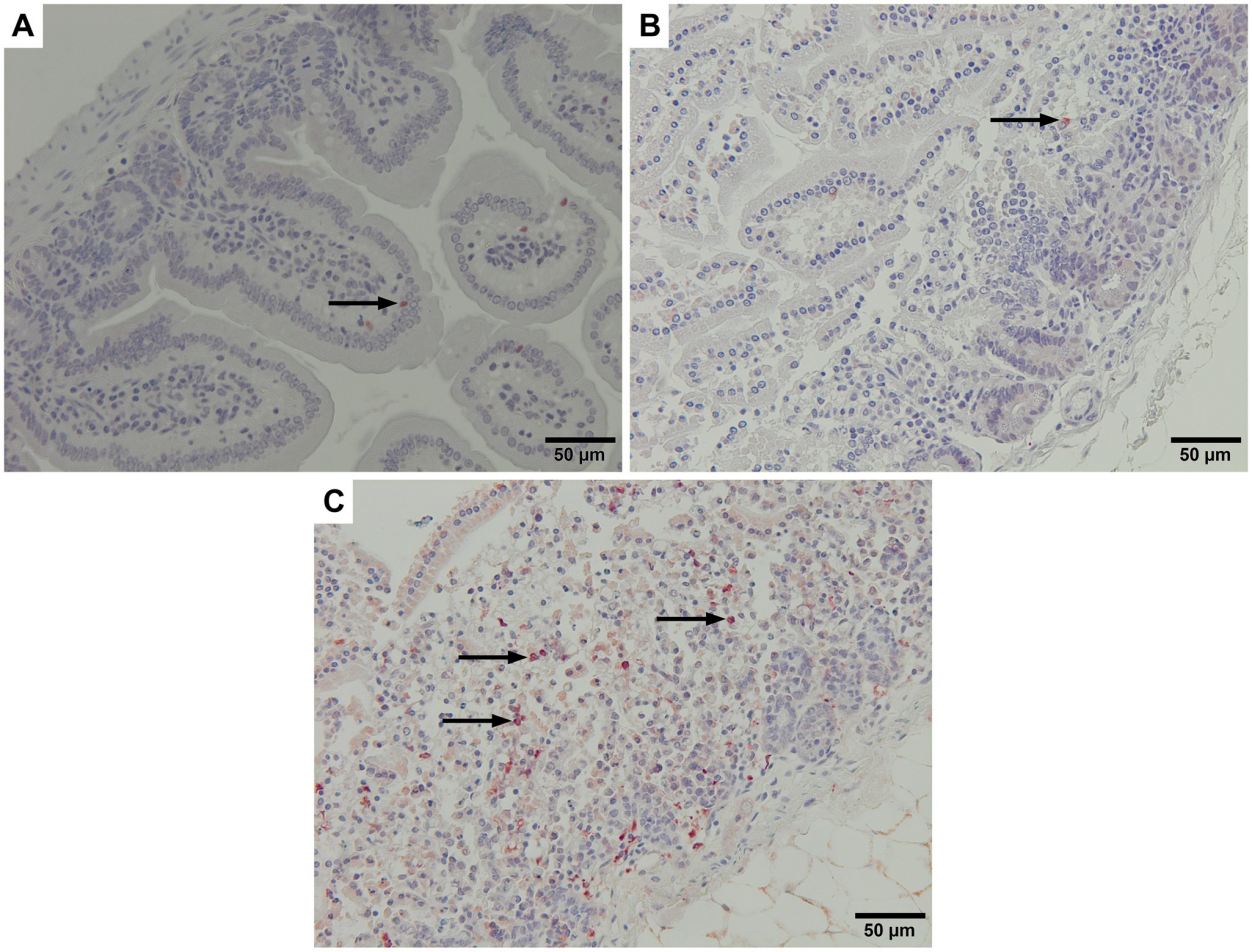
Mouse intestine subjected to the ABC method and hematoxylin counterstain. Cleaved caspase 3 in non-infected fresh intestine (A), non-infected incubated intestine (B) and infected and incubated intestine (C) all of them showing caspase 3+ cells (arrow). Specific areas of the infected and incubated intestine (C) showed a higher number of caspase 3+ cells in the lamina propria (arrows), compared with the fresh and the incubated and non-infected intestine.

Histological examination of mouse intestine samples was performed by comparing hematoxylin-eosin staining of fresh intestine samples (Fig 7A) with those incubated for 2.5 hours at 39°C, including both FhNEJ-infected and uninfected samples. Although both control (uninfected control intestines kept at the same conditions that the infected intestines) and infected intestines showed loss of villi after incubation (Fig 7B and 7C, respectively), the infected intestines showed thinner walls together with separation of the muscular and serosal layers. Detailed analysis revealed the presence of FhNEJ in the apical region and deeper areas, reaching the crypts of Lieberkühn, although no inflammatory infiltrate around FhNEJ was observed (Fig 7C and 7D). Examination of these tissues also showed the presence of pycnotic and fragmented nuclei, suggesting cellular apoptosis. This was further assessed by immunohistochemical labelling with an anti-cleaved caspase 3 antibody, whose presence was more frequently detected in certain areas of FhNEJ-infected intestine samples, compared with fresh or non-infected intestines (Fig 8). Specifically, the percentage of caspase-3 positive cells or cell debris per field (x400) examined in 12 different fields was 80.5% and 76.1% lower in fresh and non-infected intestines, respectively, compared that of infected intestines.

## Discussion

Migration through host tissues is a mechanism commonly used by the larval stages of helminth parasites. Despite the high energy and adaptive costs that this process implies, it confers evolutionary advantages and constitutes an immune evasion strategy, allowing the parasite to grow in size and modify its antigenic profile [7, 25]. In this line, FhNEJ have a dynamic composition that undergoes deep changes within the first hours post excystment, as it has been demonstrated through the establishment of *in vitro* models that include FhNEJ culture in the presence or absence of host stimuli and downstream -omics analyses [10, 26, 27]. However, none of these approaches has replicated the passage of FhNEJ through the host intestinal barrier coupled with SWATH-MS, a highly sensitive quantitative proteomics technique, which has been barely employed in the field of parasitology [12, 13]. This technique comprises two steps: an identification phase in which a protein library is constructed from all the identified peptides using a traditional HPLC-MS methodology, followed by a quantification phase in which proteins from individual samples are acquired using multiple narrow m/z windows [14]. When compared to conventional proteomic methodologies, SWATH-MS combines the sensitivity of Data-Dependent Acquisition (DDA) approaches, such as Selected Reaction Monitoring (SRM), with the high-throughput analytical capacity of Data Independent Acquisition (DIA)- based shotgun proteomics [15]. Thus, the main objective of this study was to set up an *ex vivo* model of FhNEJ intestinal crossing coupled to a quantitative proteomic comparison of these parasites before and after gut passage, by using the recently developed SWATH-MS technology. The ultimate goal of this work is to unravel the main changes in protein composition of the tegument and somatic fractions of FhNEJ driven by gut passage.

In the *ex vivo* murine model used here, FhNEJ (Italian isolate) started appearing at the extra-luminal space in as little as 30 min. This observation, together with the recovery rate of FhNEJ is similar to those made by García-Campos *et al*. using an *in vitro* model [28], in which small pieces of rat intestine were used to evaluate FhNEJ passage using the Oregon isolate (20.96% passage after 2.5 hours), although these authors described a percentage of passage at 2.5 hours of 7.44% for the Italian isolate. An additional *ex vivo* model using intestinal loops of rats non separated from the whole body and injected with FhNEJ showed comparable results to ours with an in-house obtained *F*. *hepatica* isolate (35% passage at 2.5 hours of incubation [8]). This suggests that different parasite isolates show a similar intestinal migratory behaviour, and that differences in percentage of migration could be attributed to differences in the viability of different parasite batches.

Our approach was planned to separately study a tegument-enriched fraction [10] and the somatic extract of FhNEJ after gut passage in comparison with control FhNEJ, to bring into focus the changes taking place at the host-interacting surface of the parasite, and to compare these changes with those occurring at internal tissues. Principal Component Analysis (PCA) discriminated between FhNEJ that were incubated alone and those that crossed an intestinal wall, although some intragroup variation was detected, similar to what has been described in comparable studies [29]. Annotation of proteins showed that both parasite compartments shared a high percentage of proteins, as expected. The percentage of proteins differentially regulated in each antigenic compartment was higher in the tegument than the somatic extract, indicating that the influence of the host on the regulation of protein expression mainly affects the parasite compartment that is directly exposed to host tissues.

Annotation of GO terms for each of the differentially regulated proteins revealed notable changes in metabolic and binding processes in both extracts, similar to the findings reported by Cwiklinski *et al*. [30] after GO annotation of the transcriptome of *F*. *hepatica* juveniles collected from mice 21 days post infection. The upregulated GO terms reported in our study (proteolysis, catalytic activity, transcription initiation and glutathione metabolic process) are also in agreement with those found by Cwiklinski *et al*. for FhNEJ [27], both in transcriptomic and excretory/secretory proteomic analyses of FhNEJ at several times post excystment. Conversely, we identified downregulation of TCA- and detoxification-related GO terms upon gut passage that were found to be well represented in previous proteomic and transcriptomic studies of early stages of *F*. *hepatica* (rev. in [11,30]). Nonetheless, comparison of our results with those reported by other authors could be hampered by the relative similarity between parasite stages and compartments used. Two important considerations could explain this discrepancy. First, none of the previous studies focused on profiling the proteome of *ex vivo* obtained FhNEJ after gut passage. Second, the performance of the SWATH-MS proteomic approach used in our study in terms of quantitative power is not comparable to that of semiquantitative proteomics approaches used until now. This said, our finding that TCA- related proteins are downregulated in the tegument of FhNEJ after *ex vivo* gut passage would be in line with the transition from aerobic to anaerobic metabolic states described both *in vitro* and *in vivo* in *F*. *hepatica* after FhNEJ excystment and during juvenile development (rev. in [7]). Nevertheless, it is important to mention that GO analysis could result in a broad description of the detected pathways. Notably, some reports show that the switch from aerobic to anaerobic metabolism of this parasite is a phenomenon that also occurs independently of host stimuli, given that it is detectable in the proteome of *in vitro* maintained FhNEJ 24 hours post excystment, compared with metacercariae and with FhNEJ 3 hours post excystment (e.g., [27]). Our results show that this metabolic switch seems to be enhanced by host stimulation, and that it first occurs at the host-parasite interface rather than deeper tissues of the parasite, since here TCA-related GO terms were found unaltered in the somatic extracts of *ex vivo* FhNEJ. Thus, and despite oxygen diffusion to the internal tissues of the parasite is expected to be more limited than that of its surface, we can still reason that the tegument could be the tissue ahead of the switch between aerobic and anaerobic metabolism in parasites facing the vertebrate host environment. Consistent with this assumption, REVIGO analysis of somatic extracts showed upregulation of GO terms related to gluconeogenesis and glycolysis, both linked to aerobic metabolism, similar to the observations made by Cwiklinski *et al*. [27] when comparing *in vitro* maintained FhNEJ during 3 hours and 24 hours. REVIGO analysis of both up- and downregulated GO terms in somatic extracts displayed a specific pattern different from that of the tegument. This demonstrates that proteomic changes triggered by host stimulation results in differential patterns of parasite protein regulation at the host-parasite interface, compared with those occurring in inner compartments.

It has been extensively described that CL3 and CL4 isoforms are the CL proteases mainly expressed by NEJ, while CL1, CL2 and CL5 are more abundant in adult worms (rev. in [7]). Annotation of the tegument CL isoform identified in the present study (UniProt code A0A4E0RQP0) did not manage to assign it to a specific isoform. Previously, sequence analysis of *Fasciola* cathepsin L prosegments, which regulate enzyme activity by binding to the substrate cleft, allowed for the definition of 22 amino acid consensus sequences that are specific to every CL isoform prosegment [31]. This enabled grouping of CL isoforms from CL1 to CL5 in *Fasciola*, which facilitates annotation of these proteases. When we compared the prosegment of the CL identified in our set of proteins with the abovementioned consensus sequences, we found that the 22 amino acid consensus in the prosegment sequence herein identified was almost identical to the prosegment of CL4 (90.9% identity), and less similar to the prosegment of CL1A, CL1B, CL2, CL3 and CL5 (72.7%, 81.8%, 63.6%, 40.9% and 72.7% identity, respectively; S2 Fig). Transcriptomic analysis of metacercariae and FhNEJ maintained *in vitro* for 1, 3 and 24 hours revealed high gene expression of both CL3 and CL4, which declined in juveniles collected from mice 21 days post infection and adult worms [32]. CL4 is expected to play an intracellular housekeeping function, since it has not been identified in the excretory/secretory fraction and extracellular vesicles of FhNEJ by several authors [27,32–35], with the exception of Di Maggio *et al*. [36]. These last authors identified CL4 in both secretions and somatic extracts of FhNEJ maintained in culture for 48 hours, but its presence in the secretory fraction could be attributed to contamination with tegument proteins. Noteworthy, CL3 but not CL4 has been identified in tegument extracts of FhNEJ [9]. Consequently, our results suggest that CL4 is in fact most probably restricted to tegument expression, and that this specific isoform is overexpressed in FhNEJ upon gut passage. By contrast, CL3 seems to be expressed in FhNEJ without requiring host stimulus, as it is upregulated around 1 hour after excystment in FhNEJ maintained *in vitro* [11]. Furthermore, our results and those previously reported by others suggest that CL4 in not synthesized within the gastrodermal epithelial cells of the parasite and stored in “secretory” vesicles as FhCL3 is, and raises the possibility that CL4 may also play a role at the host-parasite interface during gut penetration of the parasite. The biological relevance of CL4 expression in the intestinal phase of *F*. *hepatica* infection may have been neglected in previous studies based on *in vitro* systems that did not include host cells and/or tissues during FhNEJ incubation.

The members of the serpin superfamily have been mainly related to the regulation of peptidase activity in FhNEJ (rev. in [7]) and described as highly expressed on their surface. Here, we found three serpins over-expressed in the tegument of FhNEJ after gut passage, corresponding to the WormBase identifiers maker-scaffold10x_794_pilon-snap-gene-0.129, maker-scaffold10x_113_pilon-augustus-gene-0.45 and maker-scaffold10x_293_pilon-augustus-gene-0.19, as detailed in S1 Table. These identifiers correspond to Fh serpin 4, Fh serpin 2 and Fh serpin 5, following the phylogenetic analysis performed by [22]. More specifically, Fh serpin 2 has been observed to be associated with the apical region of the spines within the parasite tegument [37]. Fh serpin 2, which is a potent inhibitor of the small intestine protease chymotrypsin, is more abundantly transcribed in juveniles 21 days post infection than in FhNEJ maintained *in vitro* at various times post excystment and adult worms [22]. However, our results show that this molecule is overexpressed early after gut invasion by FhNEJ, suggesting that host stimulation enhances the process of Fh serpin 2 production in FhNEJ. Fh serpin 2 has also been described as an inhibitor of inflammatory-related molecules, including chymase, neutrophil elastase and cathepsin G [22], indicating that Fh serpin 2 can both protect FhNEJ during gut passage from host-derived proteolysis and play a role in immune evasion. In the present study, Fh serpin 2 overexpression was used to validate our proteomic results by immunoblot, which revealed a specific band at ~60 kDa in tegument extracts of FhNEJ that crossed the intestinal wall, but not in FhNEJ maintained *in vitro* for an equivalent time. The described molecular weight of *F*. *hepatica* native serpin 2 is ~40 kDa, although it has been shown that serpins can be found in native protein extracts complexed with other proteins or with themselves [22]. Specifically, Fh serpin 2 can form a covalent complex with chymotrypsin, resulting in the formation of a highly stable SDS complex of ~60 kDa, compatible with the band detected in our experiments. Our results confirm that Fh serpin 2 is one of the main proteins produced at the tegument of FhNEJ during gut passage, most likely to inhibit intestinal host proteases, although a role in the regulation of the activity of FhNEJ surface expressed proteases (e.g., CL4) cannot be ruled out given that Fh serpin 2 can also inhibit cathepsin L [22].

In relation to nutrition, both a low-density lipoprotein (LDL) receptor and several fatty acid binding proteins (FABPs) were found overexpressed in FhNEJ tegument after gut passage. *F*. *hepatica* is unable to synthesise lipids, and expresses several proteins for host lipid uptake [38]. This seems to be an especially active process in FhNEJ crossing the intestinal barrier, where we have found both LDL receptors and FABPs overexpressed, which could use host lipids as an essential energy source, transitioning from the use of their own endogenous energy sources to rely on host nutrients. In these very early stages, host nutrient intake still depends mainly on the tegument, since *F*. *hepatica* gastrodermal cells start their uptake activity later in the development of the parasite [39]. Additionally, both LDL receptors and FABPs have been also related to immune evasion mechanisms in *Schistosoma* and *Fasciola* [30,40].

Also linked to immune evasion due to its anti-inflammatory properties [41], and to antioxidant defence mechanisms of the parasite, glutathione-S-transferase (GST) has been extensively studied in adult worms as one of its excretory-secretory antigens, and has been identified in FhNEJ and juvenile secretions [30]. GST has been described in the cytoplasmic extensions of parenchymal cells in FhNEJ [42], which could account for its presence in the tegument of *ex vivo* obtained FhNEJ. Intriguingly, transcriptional analysis of several *F*. *hepatica* developmental stages showed that immature (21 days post-infection) flukes favour the thioredoxin-dependent antioxidant defence system instead of GST-based defence mechanisms, contrary to *F*. *gigantica* [30]. Whether GST could play a central anti-inflammatory role in the tegument of FhNEJ during gut passage, or just an accessory one (e.g., absorptive), is a matter of further investigation. In this context, immunohistological study of the intestinal fragments used in our experiments showed a lack of inflammatory infiltrate in the periphery of migrating FhNEJ, which could contribute to an overall anti-inflammatory environment in the intestine together with overexpressed Fh serpin 2 and GST in the tegument of FhNEJ. Additionally, immunostaining of intestinal fragments used in our *ex vivo* experiments showed a high number of caspase 3 positive leucocytes in the intestine after FhNEJ passage. Similar to our results, peritoneal leucocyte apoptosis driven by early stages of *F*. *hepatica* was described in sheep [43]. Thus, the lack of inflammation found in sections adjacent to FhNEJ during gut passage could also be explained by cell apoptosis.

The overexpression of the hypothetical protein D915_002431 in the tegument of *ex vivo* obtained FhNEJ is conspicuous. This protein contains the so called SEA domain, which is closely associated to regions receiving extensive O-glycosylation at the cell surface and adjacent to transmembrane proteins, including mucin-1 [24]. Glycans are rapidly shed from the tegument of FhNEJ and have important roles in invasion and tissue penetration, probably through binding to lectin receptors on host intestinal epithelial cells (rev. in [28]). Whether this specific hypothetical protein is involved in the pathogenicity and life cycle progression of *F*. *hepatica* deserves further investigation.

An additional number of proteins involved in trafficking, membrane dynamics and binding, transcriptional regulation/DNA replication and cell cycle progression were found overexpressed in the tegument of *ex vivo* obtained FhNEJ, among them several histones. The analysis of the transcriptome of *F*. *hepatica* showed that genes related to neoblasts such as histone 2A are constitutively expressed from NEJ to adult stages and have increased transcription in the juvenile parasites [27]. Additionally, regulation of histones had also been described for *Schistosoma mansoni* schistosomula maintained *in vitro*, in which expression of H2A, H2B and H4 was higher in the tegument of 5 days schistosomula, compared with 2 days and 3 hours schistosomula [44]. Similarly, a number of proteins related to cell cycle progression and cellular differentiation in early development were found overexpressed in the somatic extracts of *ex vivo* obtained FhNEJ. These proteins could be involved in the control of cell proliferation and differentiation during the intensified cell multiplication and transformation of FhNEJ towards adult worms. Overexpressed proteins in the somatic fraction of FhNEJ also included the putative vacuolar protein sorting vps26. This protein has been described as one of the main components of the retromer complex, which is involved in the trans-Golgi network that packages proteins into vesicles destined to lysosomes, secretory vesicles, or the cell surface. Notably, a similar protein was described in *Entamoeba histolytica* and was related to trafficking of cysteine proteases in this parasite [45], and it has similarly been linked to vesicular protein sorting and biogenesis of secretory organelles in *Plasmodium falciparum* and *Toxoplasma gondii* [46,47]. Vps26 could also be participating in vesicular trafficking in FhNEJ and could therefore be of crucial importance for the survival and development of the parasite at early stages of infection.

Regarding downregulated proteins, it has been described that HSP-90 and alpha crystallin are more highly expressed in metacercariae and FhNEJ maintained *in vitro* 1 hour after excystment compared to FhNEJ at 3 and 24 hours post excystment [27]. These proteins have been associated with the response to sudden environmental changes encountered by “dormant” stages of different parasites. Our results show that protection against reactive oxygen species of the host by HSPs could be crucial for FhNEJ found within the intestinal lumen, and may be put on the back burner once FhNEJ have passed through the intestinal wall.

In summary, we have shown that host stimulation (gut passage) triggers changes in the proteome of FhNEJ both at the tegument and somatic levels, which results in the expression of defined sets of proteins as well as an acceleration in the expression of additional proteins that would otherwise occur at a slower rate in FhNEJ axenically maintained *in vitro*. Our proteomic findings of upregulated and downregulated expressed proteins in the two fractions are similar to previous reports, as above-mentioned for each protein or group of proteins. Noteworthy, Hanna and Jura [48] described that significantly more *F*. *gigantica* flukes successfully established in mice via intraperitoneal injection of NEJ than oral infection with metacercariae. The authors attributed these differences to inappropriate physicochemical conditions for excystment in the gut of the mouse, although the mouse model has been considered suitable for studies on migration and establishment of *F*. *gigantica* by several authors (e.g., [49]). Whether these differences are also true for *F*. *hepatica* is a matter of future investigations and could contribute to decipher the importance of intestinal passage in the physiology and establishment of *F*. *hepatica* in the vertebrate host.

## Supporting information

S1 Fig REVIGO analysis of regulated proteins. The analysis shows the enriched GO terms in the Biological Process (BP) category, for upregulated (green) and downregulated (pink) proteins annotated in somatic and tegument extracts of FhNEJ after gut passage, compared with control FhNEJ. Size of each circle represents the relative abundance of each BP term. Performed at http://revigo.irb.hr/.
(TIF)

S2 Fig Comparison of the consensus amino acid sequence of the prosegment of cathepisns L1 to L5 with CL_A0A4E0RQP0. Alignment of the consensus sequences of the non-conserved *Fasciola* cathepsin L (CL1A, CL1B, CL2, CL3, CL4 and CL5) protease prosegment C-terminal regions, as described in [31], and the cathepsin L sequence identified as over-expressed in the tegument of FhNEJ after gut passage (CL_A0A4E0RQP0) in shown. As shown in the figure, the cathepsin L found in our study shows the highest percentage of identity in this region (90.9%) with the consensus sequence of CL4. Gaps in the alignment are represented by a point.
(TIF)

S1 Video Intestinal passage of FhNEJ. The video shows FhNEJ crossing the intestine in the *ex vivo* model used in our experiments.
(AVI)

S1 Table Up- and down-regulated proteins in FhNEJ upon intestinal passage. Details on annotation and analysis of upregulated and downregulated proteins in both tegument and somatic extracts are shown, including Protein ID, Uniprot Accession number, Description, Log2FC and GO IDs for Cellular component (CC), Molecular function (MF) and Biological process (BP). Teg up: proteins upregulated in FhNEJ tegument; Teg down: proteins downregulated in FhNEJ tegument; Som up: proteins upregulated in FhNEJ somatic extract; Som down: proteins downregulated in FhNEJ somatic extract.
(XLSX)
