# Supplementary figures and images for: Study of the migration of Fasciola hepatica juveniles across the intestinal barrier of the host by quantitative proteomics in an ex vivo model
Source: PLoS Negl Trop Dis. 2022 Sep 16;16(9):e0010766. doi: 10.1371/journal.pntd.0010766 (PMC9518905; doi:10.1371/journal.pntd.0010766)

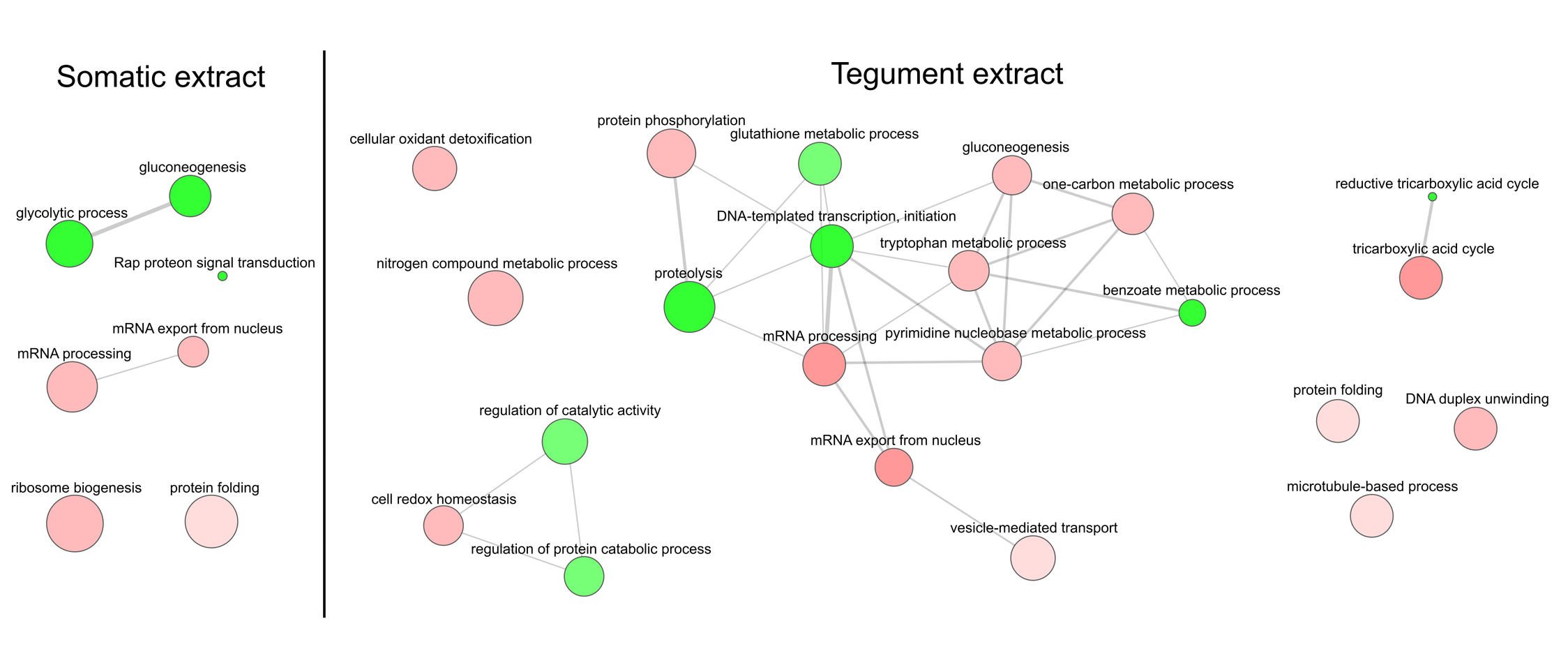

Supplement: S1 Fig — The analysis shows the enriched GO terms in the Biological Process (BP) category, for upregulated (green) and downregulated (pink) proteins annotated in somatic and tegument extracts of FhNEJ after gut passage, compared with control FhNEJ. Size of each circle represents the relative abundance of each BP term. Performed at http://revigo.irb.hr/. (TIF) [file pntd.0010766.s001.tif]

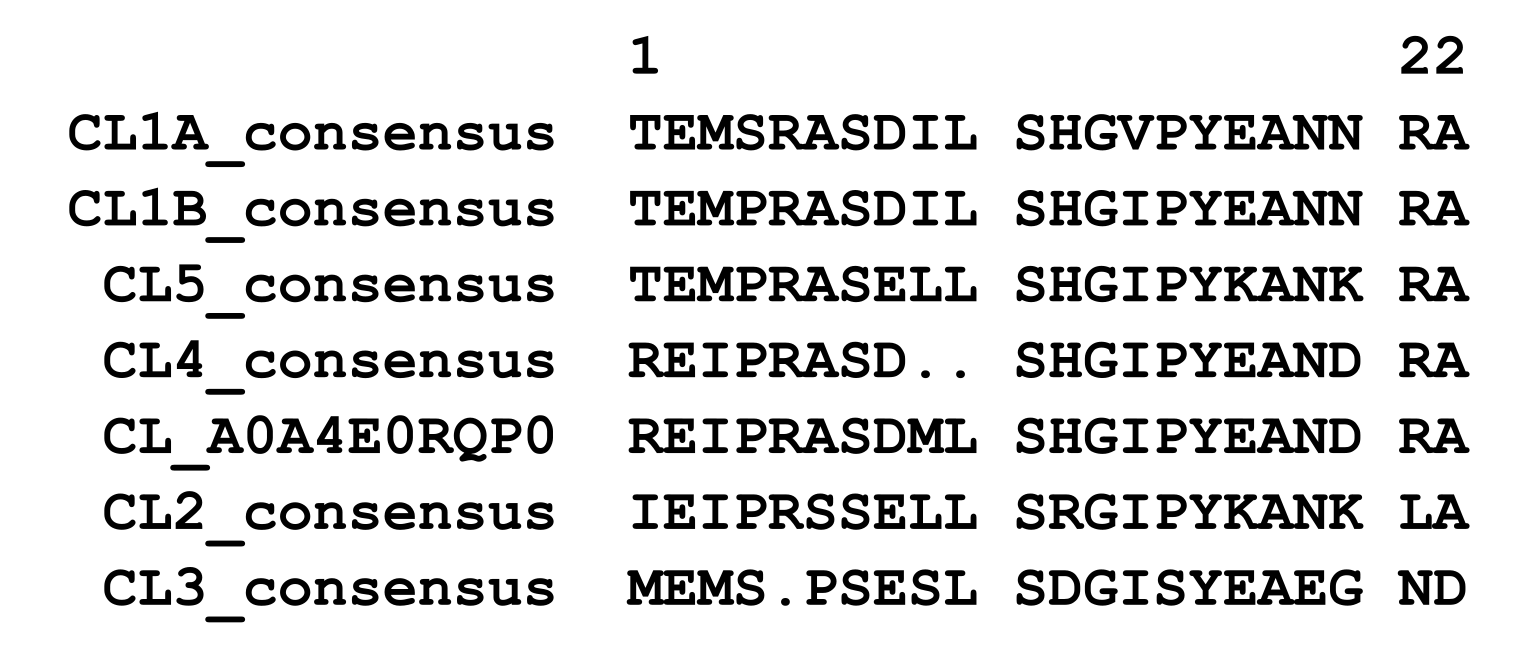

Supplement: S2 Fig — Alignment of the consensus sequences of the non-conserved Fasciola cathepsin L (CL1A, CL1B, CL2, CL3, CL4 and CL5) protease prosegment C-terminal regions, as described in [31], and the cathepsin L sequence identified as over-expressed in the tegument of FhNEJ after gut passage (CL_A0A4E0RQP0) in shown. As shown in the figure, the cathepsin L found in our study shows the highest percentage of identity in this region (90.9%) with the consensus sequence of CL4. Gaps in the alignment are represented by a point. (TIF) [file pntd.0010766.s002.tif]
